# Editorial: Host-Microbiome Interactions and Influence on Performance During Acute Environmental, Nutritional, Physical, and Cognitive Stress, Volume II

**DOI:** 10.3389/fphys.2022.894922

**Published:** 2022-04-08

**Authors:** Sarah C. Pearce, J. Philip Karl, Nicholas C. Zachos

**Affiliations:** ^1^ National Laboratory for Agriculture and the Environment, USDA-ARS, Ames, IA, United States; ^2^ Military Nutrition Division, US Army Research Institute of Environmental Medicine, Natick, MA, United States; ^3^ Department of Medicine, Johns Hopkins University School of Medicine, Baltimore, MD, United States

**Keywords:** microbiome, intestine, stress, physiology, host

The gastrointestinal (GI) tract ensures the digestion and absorption of nutrients and modulates host metabolism and physiology. The GI tract is also home to the gut microbiota, a diverse and dynamic microbial community that includes bacteria, viruses, yeast and other fungi. The gut microbiota assists in digestion, produces vitamins, defends against foreign pathogens, provides a protective barrier for the intestinal epithelium, signals the brain, and influences immune function. Thus, the relationship between intestinal function and the gut microbiota is bi-directional. Various acute stressors including exercise, sleep deprivation, thermal stress, high altitude, toxin exposures, psychological stress and dietary changes can impact this dynamic relationship by altering normal host intestinal function and/or the composition and metabolic activity of the gut microbiota (Karl et al., 2018). This implies that the effects of stress on human performance may be mediated, in part, by the bi-directional interaction between the host intestinal mucosa and the gut microbiota.

This research topic solicited studies that advance current understanding of how environmental, nutritional, physical, and cognitive stressors alter intestinal function, the gut microbiota and their interactions. The two volumes within the topic include seven original research publications, three review articles, and one brief research report. Collectively, these papers provide novel information on how gut barrier function is altered in disease, the effects of environmental and psychological stress on host-gut microbiota interactions, and the potential for specific nutrients and microbially-derived interventions to beneficially modulate host-microbiota interactions.

Two narrative reviews on gut barrier function set the stage for Volumes I and II. One focuses on the role of tight junctions in disease and the potential positive influence of phytochemicals in maintaining intestinal integrity by Panwar et al., while the second considers the role of hypoxia-inducible factor 1-α in maintaining intestinal homeostasis and intestinal integrity by Kumar et al. (2020). Panwar et al. reviews the critical functions of junctional proteins within the context of metabolic and autoimmune diseases including inflammatory bowel diseases with a specific focus on the role of plant phytochemicals such as quercetin and curcumin for modulating tight junction proteins. Kumar et al. (2020), discusses the regulation and function of HIF-1α, how both the mucous layer and tight junctions are regulated by HIF-1α, and the potential role of NF-KB/HIF-1α crosstalk in inflammatory diseases. These reviews highlight the therapeutic potential of natural compounds derived from herbal products as well as targeting HIF-1α to enhance intestinal barrier function *via* regulating tight junctions.

 The other papers within Volumes I and II provide novel insights into effects of environmental, physical and psychological stress on host-gut microbiome interactions, while highlighting the integral role of gut microbiome-intestinal barrier crosstalk in mediating effects of stress on host health and performance. Environmental and physical stressors examined include foodborne pathogens (Stamps et al.), chemical exposure (Kimono et al., 2019), and simulated spaceflight (Šket et al., 2020). All these studies identified gut microbiota or associated metabolites that may mediate host responses to these stressors. For example, within a randomized controlled trial where prophylaxis with the antibiotic rifaximin was ineffective (Rimmer et al., 2018), Stamps et al. correlated increased gut microbiota diversity with protection against gastrointestinal illness caused by *Campylobacter jejuni*, a common diarrhea-causing foodborne pathogen. Gut microbiota diversity is also identified as a feature mediating susceptibility to illness and disease by Wang et al. (2020a) who examine gut microbial signatures associated with urticaria and by Xiao et al. who review gut microbes and metabolites associated with irritable bowel syndrome. Kimono et al. (2019) show that ingesting pyridostigmine bromide and permethrin, both chemicals used during the Gulf War, increases fecal levels of bacterially derived pathogen-associated molecular patterns in mice leading to activation of enteric glial cells, oxidative stress, colonic inflammation and intestinal barrier permeability. These findings implicate that disrupted microbiota and altered intestinal barrier function responsible for underlying Gulf War Illness pathology. Finally, Šket et al. (2020) examine the independent and combined effects of hypoxia and physical inactivity, documenting rapid inactivity-induced changes in gut microbiota derived metabolites, including acetate, formate and hippurate, which may contribute to long-term physical and mental outcomes such as metabolic disease and depression, respectively. Moreover, this study also provides novel insight into the time course and complexity of host-gut microbiota interactions previously associated with systemic decrements in host health and physiology during simulated spaceflight (Šket et al., 2017a; Šket et al., 2017b).

Studies examining effects of psychological stressors also expand our understanding of gut microbiota-intestinal barrier interactions beyond the gut by using rodent models to probe dynamics of the gut microbiota-gut-brain axis. Hoke et al. use a previously validated social stress paradigm that causes post-traumatic stress disorder-like symptomology and related decrements in metabolic and psychiatric health in mice (Hammamieh et al., 2012; Chakraborty et al., 2015; Gautam et al., 2015; Muhie et al., 2017) to show that those decrements may be associated with stress-induced changes in intestinal barrier function and in composition and bioenergetics of the gut microbiota. Relatedly, Wei et al. (2019) use chronic unpredictable mild stress, a model that induces depressive and anxiety-like behavior in rats, to show that alterations in gut microbiota composition, increased intestinal permeability and bacterial invasion into the colonic mucous layer may comprise one pathway linking chronic psychological stress to colonic inflammation.

Collectively, these studies reinforce the concept that environmental, physical and psychological stressors can directly alter gastrointestinal physiology and/or gut microbiota community composition and metabolic activity initiating a cycle leading to degradations in gut barrier function, modulation of the gut microbiota, and, ultimately, impaired health and performance (Karl et al., 2018). However, those relationships also imply that interventions targeting the gut microbiota and/or intestinal barrier may provide countermeasures for ameliorating stress-induced decrements in host health and performance. That concept is addressed in two studies within Volumes I and II. In one study, Foxx et al. (2021) examine immunization with *Mycobacterium vaccae,* an environmentally derived bacterium with immune-regulatory and anti-inflammatory properties (Zuany-Amorim et al., 2002), and expand evidence that this bacterium may ameliorate stress-induced impairments in cognitive performance (Reber et al., 2016) using a “two-hit” rodent stress model consisting of chronic circadian disruption followed by acute social defeat, immunization with *M. vaccae* NCTC 11659 is shown to stabilize the gut microbiota of circadian disrupted mice but improve behavioral and cognitive responses to social defeat only in mice chronically exposed to normal light/dark cycles and not those subjected to circadian disruption. Wang et al. (2020b) use a more traditional probiotic-based approach to show strain specific effects of *Lactobacillus rhamnosus* on gastrointestinal motility in a rodent model of constipation. Taken together, these two studies add to a rapidly expanding base of evidence supporting microbially-derived interventions for optimizing stress responses but reinforce the concept that the effectiveness of these countermeasures may be specific to the stress exposure and strain of microorganism (Hill et al., 2014; Agans et al., 2020).

Collectively, the eleven papers published within this research topic highlight the diverse aims and broad applications of research within the field, and contribute to advancing understanding of how acute environmental, nutritional, physical, and cognitive stress influence host-gut microbiota interactions within the GI tract and the resulting impact on other organ systems. Further, the identification of potential intervention strategies for beneficially modulating those interactions underscore the potential impact of future research within the field for improving host responses to and performance under stress.
